# Memory consolidation circuits and the glymphatic system in sleep

**DOI:** 10.1002/alz.089231

**Published:** 2025-01-03

**Authors:** Valeria Vasciaveo, Antonino Casile, Alma Ferro, Giulia Morello, Elena Tamagno, Michela Guglielmotto

**Affiliations:** ^1^ University of Torino, Torino Italy; ^2^ Neuroscience Institute Cavalieri Ottolenghi, Orbassano, Torino Italy; ^3^ University of Camerino,, Camerino Italy; ^4^ Neuroscience Institute Cavalieri Ottolenghi, Orbassano Italy; ^5^ Neuroscience Institute Cavalieri Ottolenghi, Torino Italy; ^6^ University of Turin, Turin, Torino Italy; ^7^ University of Torino, Torino, Torino Italy

## Abstract

**Background:**

Understanding the neuronal mechanisms of learning and memory is one of the major goals in neurophysiology and neuropsychology. Disorders related to memory consolidation are often the consequences of dynamic plasticity changes, which may lead to a reduction in spine number and density, impairing neural networks. Sleep is one of the major physiological prerequisites for memory consolidation, especially during NREM sleepwhen glymphatic system clearance takes place, too.

**Method:**

We recently validated a protocol of SF, which mimics more closely than sleep deprivation a typical impaired sleep behavior in psychiatric and neurological disorders. In this project we want to extend our previously study on the WT mouse model to unravel the mechanisms responsible for glymphatic system disruption and its correlation with memory circuitry malfunction. We will investigate which neuronal population is affected by SF by considering three regions involved in memory consolidation: hippocampus, prefrontal cortex, and the amygdala. Additionally, chronic EEG‐recordings during natural sleep/wake behavior will be performed in freely behaving mice, and all temporal and spectral parameters involved in memory consolidation will be analyzed. As for glymphatic system, we will study through MRI the possible glymphatic flux alteration with Gd‐DTPA as MRI tracer in the three interested regions.

**Result:**

In addition to the proof that SF accelerates Alzheimer’s Disease (AD) pathology, we observed interesting modifications of cognitive abilities in wild type (WT) mice during behavioral tests. Moreover, in both the 5xFAD mouse model for AD and the corresponding WT mice after SF, we detected an aquaporin‐4 (AQP4) modulation, which is known to play a role in neurological diseases by disrupting the glymphatic system flux. Our final goal will be to understand which is the specific circuit that leads to memory impairment, and what happens to the neurons involved during SF: degeneration, excitotoxicity or astrocyte communication failure. A future study could be to test the ability to restore cognitive functions by letting the mice free to sleep one month after SF protocol.

**Conclusion:**

Thanks to this project, we go back to the bench to find the correlation never observed before between memory circuit deficits and the possible involvement of glymphatic system during SF.

